# Supplementary material for: Human local field potentials in motor and non-motor brain areas encode upcoming movement direction
Source: Commun Biol. 2024 Apr 27;7:506. doi: 10.1038/s42003-024-06151-3 (PMC11055917; doi:10.1038/s42003-024-06151-3)
Supplement: Supplementary file 2 — Supplementary Material [file 42003_2024_6151_MOESM2_ESM.pdf]

Subject	x	y	z
Subject 1	-9,090909004	6,124593735	44,94412231
	-12,62626266	6,124593735	44,94412231
	-39,39393997	6,124593735	44,94412231
	-42,92929459	6,124593735	44,94412231
	-46,46464539	6,124593735	44,94412231
	-50,50505066	6,124593735	44,94412231
	-7,575757504	45,30327606	24,15659523
	-13,13131332	45,81809616	24,18375778
	-28,28282738	46,33291626	24,21092033
	-32,32323074	46,33291626	24,21092033
	-35,85858536	46,33291626	24,21092033
	-39,39393997	46,33291626	24,21092033
	-5,555555344	22,13595963	33,81742096
	-9,090909004	22,13595963	33,81742096
	-24,24242401	22,13595963	33,81742096
	-28,28282738	22,13595963	33,81742096
	-32,32323074	22,13595963	33,81742096
	-43,43434525	22,13595963	33,81742096
	-47,4747467	22,13595963	33,81742096
	-51,51515198	22,13595963	33,81742096
	-4,040403843	-13,07874012	58,07904434
	-8,080807686	-13,07874012	58,07904434
	-12,12121201	-13,07874012	58,07904434
	-30,80808067	-13,13026524	59,16464233
	-34,34343338	-13,13026524	59,16464233
	-38,38383865	-13,13026524	59,16464233
	-42,42424393	-13,13026524	59,16464233
	-45,95959473	-13,13026524	59,16464233
	-49,49494934	-13,13026524	59,16464233
	33,33333206	20,54126358	2,172101736
	36,86868668	20,54126358	2,172101736
	40,40404129	20,54126358	2,172101736
	44,44444275	20,54126358	2,172101736
	48,48484802	20,54126358	2,172101736
	6,060606003	47,62104034	-3,207987547
	10,10101032	47,62104034	-3,207987547
	14,14141369	47,62104034	-3,207987547
	29,2929287	47,62104034	-3,207987547
	32,8282814	47,62104034	-3,207987547
	36,36363602	47,62104034	-3,207987547
	40,40404129	47,62104034	-3,207987547

	44,44444275	47,64680099	-3,802481651
	5,050505161	43,3470459	21,87674904
	8,585858345	43,3470459	21,87674904
	12,12121201	43,3470459	21,87674904
	23,73737335	43,3470459	21,87674904
	27,27272797	43,3470459	21,87674904
	31,31313133	43,3470459	21,87674904
	35,3535347	43,3470459	21,87674904
	38,88888931	43,3470459	21,87674904
	42,42424393	43,3470459	21,87674904
	46,46464539	43,3470459	21,87674904
	30,30303001	20,84955406	17,42480087
	34,34343338	20,84955406	17,42480087
	37,87878799	20,84955406	17,42480087
	41,41414261	20,84955406	17,42480087
	45,45454407	20,84955406	17,42480087
	49,49494934	20,84955406	17,42480087
	53,03030396	20,84955406	17,42480087
	56,56565475	20,84955406	17,42480087
	38,88888931	7,978631973	27,62887955
	42,42424393	7,978631973	27,62887955
	46,46464539	7,978631973	27,62887955
	50,50505066	7,978631973	27,62887955
	54,54545593	7,978631973	27,62887955
	58,08080673	7,978631973	27,62887955
	6,060606003	5,507153988	36,20501328
	10,10101032	5,507153988	36,20501328
	13,63636398	5,507153988	36,20501328
	36,36363602	5,507153988	36,20501328
	40,40404129	5,507153988	36,20501328
	44,44444275	5,507153988	36,20501328
	47,97979736	5,507153988	36,20501328
	51,51515198	5,507153988	36,20501328
	4,040403843	49,00963593	33,05868149
	8,080807686	49,00963593	33,05868149
	11,61616135	49,00963593	33,05868149
	15,15151501	49,00963593	33,05868149
	19,19191933	49,03539658	32,51588058
	23,23232269	49,06116104	31,9730835
	27,27272797	49,06116104	31,9730835
	30,80808067	49,06116104	31,9730835
	3,030303001	25,79079247	43,80510712

	7,070706844	25,79079247	43,80510712
	11,11111069	25,79079247	43,80510712
	22,22222137	25,79079247	43,80510712
	26,26262665	25,79079247	43,80510712
	30,30303001	25,79079247	43,80510712
	33,83838272	25,79079247	43,80510712
	37,37373734	25,79079247	43,80510712
	41,41414261	25,79079247	43,80510712
	45,45454407	25,79079247	43,80510712
	48,98989868	25,79079247	43,80510712
	2,020201921	7,617098808	56,99435425
	6,060606003	7,617098808	56,99435425
	10,10101032	7,617098808	56,99435425
	13,63636398	7,617098808	56,99435425
	17,17171669	7,617098808	56,99435425
	21,21212196	7,617098808	56,99435425
	25,25252533	7,617098808	56,99435425
	28,78787804	7,617098808	56,99435425
	32,32323074	7,617098808	56,99435425
	36,36363602	7,617098808	56,99435425
	40,40404129	7,617098808	56,99435425
**Subject 2**	7,777777672	-45,82024384	30,2319603
	12,02020168	-45,82024384	30,2319603
	58,08080673	-45,82024384	30,2319603
	62,62626266	-45,82024384	30,2319603
	41,41414261	-28,9947834	1,952808857
	45,45454407	-28,9947834	1,952808857
	61,61616135	-28,9947834	1,952808857
	65,65656281	-28,9947834	1,952808857
	69,69696808	-28,9947834	1,952808857
	29,2929287	-28,39709473	-11,65347481
	33,33333206	-28,39709473	-11,65347481
	49,49494934	-28,39709473	-11,65347481
	61,61616135	-28,39709473	-11,65347481
	65,65656281	-28,39709473	-11,65347481
	39,39393997	3,141731501	-36,76939392
	43,43434525	3,141731501	-36,76939392
	47,4747467	3,141731501	-36,76939392
	51,51515198	3,141731501	-36,76939392
	55,55555725	3,141731501	-36,76939392
	8,030303001	41,03186035	-17,89234161
	12,62626266	41,03186035	-17,89234161

	17,17171669	41,03186035	-17,89234161
	38,38383865	41,03186035	-17,89234161
	42,42424393	41,03186035	-17,89234161
	46,96969604	41,03186035	-17,89234161
	44,44444275	33,96882629	1,988138676
	48,48484802	33,96882629	1,988138676
	52,5252533	33,96882629	1,988138676
	56,56565475	33,96882629	1,988138676
	42,42424393	11,73737431	13,67907906
	46,46464539	11,73737431	13,67907906
	62,62626266	11,73737431	13,67907906
	66,66666412	11,73737431	13,67907906
	9,191919327	21,6730938	21,82151794
	13,63636398	21,6730938	21,82151794
	51,51515198	21,6730938	21,82151794
	56,06060791	21,6730938	21,82151794
	60,60606003	21,6730938	21,82151794
	11,81818199	58,63277054	4,290693283
	16,16161537	58,63277054	4,290693283
	20,20202065	58,63277054	4,290693283
	24,24242401	58,63277054	4,290693283
	28,28282738	58,63277054	4,290693283
	32,32323074	58,63277054	4,290693283
	44,94949341	58,63277054	4,290693283
	49,49494934	58,63277054	4,290693283
	7,575757504	43,70772171	14,27756214
	11,91919231	43,70772171	14,27756214
	16,16161537	43,70772171	14,27756214
	33,33333206	43,70772171	14,27756214
	50,50505066	43,70772171	14,27756214
	54,54545593	43,70772171	14,27756214
	59,09090805	43,70772171	14,27756214
	12,62626266	25,56150818	-18,19033241
	13,63636398	27,46621132	-14,50436878
	26,76767731	44,71417618	14,82040691
	27,77777863	46,64206696	17,69733238
	29,2929287	48,57253265	20,51997757
	30,80808067	50,4772377	23,88542175
	5,555555344	39,17696381	22,74504662
	9,898989677	39,17696381	22,74504662
	14,14141369	39,17696381	22,74504662
	35,3535347	39,17696381	22,74504662

	39,39393997	39,17696381	22,74504662
	43,93939209	39,17696381	22,74504662
	48,48484802	39,17696381	22,74504662
	52,5252533	39,17696381	22,74504662
	9,04040432	34,18248367	40,98291397
	13,13131332	34,18248367	40,98291397
	17,17171669	34,18248367	40,98291397
	21,21212196	34,18248367	40,98291397
	25,75757599	34,18248367	40,98291397
	30,30303001	34,18248367	40,98291397
	46,46464539	34,18248367	40,98291397
	50,50505066	34,18248367	40,98291397
	9,696969986	34,07943344	43,15410995
	13,13131332	34,49120331	45,35246658
	16,66666603	35,41779327	47,57799149
	19,69696999	36,34438324	49,80351257
	6,919191837	4,528781891	41,59497833
	11,060606	4,528781891	41,59497833
	35,3535347	4,528781891	41,59497833
	39,39393997	4,528781891	41,59497833
	43,43434525	4,528781891	41,59497833
	56,56565475	4,528781891	41,59497833
	5,050505161	-0,5684918761	55,47415543
	9,090909004	-0,5684918761	55,47415543
	33,33333206	-0,5684918761	55,47415543
	37,37373734	-0,5684918761	55,47415543
	41,41414261	-0,5684918761	55,47415543
	45,45454407	-0,5684918761	55,47415543
**Subject 3**	-5,707070827	31,79086876	-19,02232742
	-6,868686676	33,72133636	-15,93085766
	-10,40404034	39,46378708	-5,526911736
	-11,61616135	41,8910408	-1,989547133
	-19,69696999	55,80577087	20,3574543
	-20,7070713	57,73623657	23,18010139
	-3,131313086	45,73694611	-7,369545937
	-6,717171669	45,73694611	-7,369545937
	-21,21212196	45,73694611	-7,369545937
	-24,74747467	45,73694611	-7,369545937
	-38,88888931	45,73694611	-7,369545937
	-42,42424393	45,73694611	-7,369545937
	-3,131313086	37,62263107	11,99753475
	-6,717171669	37,62263107	11,99753475

	-10,30303001	37,62263107	11,99753475
	-31,81818199	37,62263107	11,99753475
	-42,42424393	37,62263107	11,99753475
	-46,46464539	37,62263107	11,99753475
	-30,80808067	-20,95260429	-16,70632935
	-34,34343338	-20,95260429	-16,70632935
	-52,5252533	-20,95260429	-16,70632935
	-56,06060791	-20,95260429	-16,70632935
	13,13131332	30,65818024	-16,70384979
	13,63636398	32,56288528	-13,01788616
	16,16161537	40,19638443	1,245891571
	20,7070713	53,51462936	25,13399506
	21,21212196	55,41933441	28,49944115
	4,444444656	41,70082855	-9,509923935
	7,929293156	41,70082855	-9,509923935
	21,71717262	41,70082855	-9,509923935
	25,25252533	41,70082855	-9,509923935
	38,88888931	41,70082855	-9,509923935
	42,42424393	41,70082855	-9,509923935
	45,95959473	41,70082855	-9,509923935
	2,979798079	26,51214218	28,60673332
	6,515151501	26,51214218	28,60673332
	24,24242401	26,51214218	28,60673332
	41,91919327	26,51214218	28,60673332
	45,45454407	26,51214218	28,60673332
	49,49494934	26,51214218	28,60673332
	9,191919327	2,006379843	36,02030945
	31,31313133	2,006379843	36,02030945
	42,42424393	2,006379843	36,02030945
	53,53535461	2,006379843	36,02030945
	4,343434334	33,10131836	42,01418686
	7,97979784	33,10131836	42,01418686
	11,46464634	33,10131836	42,01418686
	15,15151501	33,10131836	42,01418686
	29,79797935	33,10131836	42,01418686
	33,33333206	33,10131836	42,01418686
	37,37373734	33,10131836	42,01418686
	40,90909195	33,10131836	42,01418686
	4,646464825	10,60296726	59,32852554
	8,232323647	10,60296726	59,32852554
	19,19191933	10,60296726	59,32852554
	22,72727203	10,60296726	59,32852554

	33,33333206	10,60296726	59,32852554
	36,86868668	10,60296726	59,32852554
	35,3535347	4,325774193	-35,5090065
	38,88888931	4,325774193	-35,5090065
	42,42424393	4,325774193	-35,5090065
	45,95959473	4,325774193	-35,5090065
	49,49494934	4,325774193	-35,5090065
	21,71717262	-11,32517529	-24,49375534
	24,74747467	-11,32517529	-24,49375534
	34,84848404	-11,32517529	-24,49375534
	37,87878799	-11,32517529	-24,49375534
	40,90909195	-11,32517529	-24,49375534
	22,22222137	-26,28113937	-12,84236431
	25,75757599	-26,28113937	-12,84236431
	29,2929287	-26,28113937	-12,84236431
	33,33333206	-26,28113937	-12,84236431
	54,54545593	-26,28113937	-12,84236431
	58,08080673	-26,28113937	-12,84236431
	21,71717262	-23,68084908	-25,2077446
	31,81818199	-23,68084908	-25,2077446
	34,84848404	-23,68084908	-25,2077446
	37,87878799	-23,68084908	-25,2077446
	51,51515198	-23,68084908	-25,2077446
	55,55555725	-23,68084908	-25,2077446
**Subject 4**	5,85454607	24,226717	-30,42224884
	6,221520424	26,68647766	-26,65895844
	8,977379799	45,67848206	2,246495962
	9,309588432	47,96646118	5,751360416
	9,638191223	50,22862625	9,231609344
	3,148604155	-25,71803093	58,57487106
	14,78814125	-25,93608284	58,4523201
	18,6564579	-26,01051521	58,39228821
	37,79940796	-26,37899399	57,90554047
	41,58029556	-26,44677734	57,77402496
	45,34593201	-26,5104084	57,6353035
	3,132441759	-9,858945847	60,73404312
	14,66420364	-10,95115852	61,0719223
	18,4916935	-11,31983948	61,16516113
	22,30672264	-11,6906662	61,24627304
	26,10834885	-12,06317806	61,31476212
	37,4250145	-13,18248272	61,44393158
	41,16941833	-13,55352783	61,46692657

	5,411727905	13,49992657	55,33231735
	16,88385582	12,16689491	55,63965988
	20,69262695	11,71068382	55,7224617
	24,48995209	11,24807167	55,79253006
	28,27589417	10,78005028	55,8496933
	32,04961395	10,30766582	55,89376831
	35,81001282	9,831617355	55,92508316
	39,55744553	9,353945732	55,94560623
	43,29343033	8,876457214	55,95845032
	9,364928246	9,756391525	41,55205536
	11,1479845	10,47151375	45,04194641
	16,38181877	12,52762032	55,24727631
	18,08589745	13,18971443	58,57074356
	19,77168465	13,84587002	61,86188126
	8,316105843	28,44685555	48,85814285
	12,13099957	27,91650391	48,80686188
	15,94166851	27,37756729	48,74668884
	19,74645805	26,83024216	48,67648697
	23,54395676	26,27411842	48,59499359
	27,33236313	25,70854187	48,50062561
	31,110075	25,13330078	48,39294815
	34,87623978	24,54917336	48,27276993
	38,63015366	23,95867729	48,14093018
	42,37174225	23,36386681	47,99967575
	46,10172272	22,76714897	47,8526001
	8,848270416	35,81494904	37,20889282
	11,76971912	36,91020584	39,4344635
	14,66745377	37,98823547	41,63259125
	17,54328918	39,05179977	43,80597687
	20,39893723	40,10277176	45,95655823
	5,48180151	40,98947144	32,99885178
	17,04043198	40,10252762	33,59853363
	20,8698349	39,78293991	33,77617264
	32,27100754	38,74322891	34,23422241
	36,04018402	38,37178802	34,36322021
	39,79458237	37,99141693	34,48369217
	27,06120491	27,39079285	-23,37637901
	27,12170219	28,59597588	-19,02048683
	27,45758057	37,8272171	15,27062607
	27,47212601	38,90419006	19,44326782
	27,47667122	39,95313644	23,57073212
	27,47109985	40,97195435	27,64485168

	27,45583725	41,96123505	31,66157341
	6,78899622	3,513105869	33,24496078
	10,72668839	3,263277769	33,52306747
	30,25837135	1,936570168	34,75862503
	34,11064148	1,653722644	34,97274399
	37,9392662	1,365746617	35,17734909
	41,74240875	1,0749861	35,37309265
	45,51985931	0,7821388245	35,56107712
	49,27403641	0,4889966547	35,74412155
	53,00816345	0,1974937916	35,92505646
	6,046336651	30,71377754	16,94434357
	33,22212601	28,89465523	17,63469315
	37,02058029	28,58863068	17,69667435
	40,79515839	28,27523804	17,75464821
	3,539314032	32,73523331	-4,064940929
	34,59460449	31,77501297	-2,885588884
	45,87200928	31,26237679	-2,461021423
	38,8552742	-9,013586044	19,72097397
	50,29806137	-8,559112549	19,24967766
	54,06134033	-8,410950661	19,09161568
	57,80467606	-8,26346302	18,93433571
	61,53314972	-8,115442276	18,77869987
	65,25367737	-7,966706276	18,62642479
	37,67757034	3,891815901	10,06772709
	41,46741867	3,29564786	10,32369995
	52,70714951	1,494168758	11,09312248
	56,41947174	0,892336607	11,35049915
	60,12136841	0,2921441197	11,60844803
	33,54728317	8,961251259	2,385173321
	37,38097382	8,662311554	2,422833681
	52,47926331	7,426642895	2,601127386
	56,2094841	7,109527588	2,652225494
	0	67	-4,212999821
	0	-22,62000084	75
	7,575757504	-51,79198074	25,56361771
	11,11111069	-51,79198074	25,56361771
	14,64646435	-51,79198074	25,56361771
	49,49494934	-51,79198074	25,56361771
**Subject 5**	53,03030396	-51,79198074	25,56361771
	56,56565475	-51,79198074	25,56361771
	60,10100937	-51,79198074	25,56361771
	63,63636398	-51,79198074	25,56361771

	37,87878799	10,29553509	22,30953979
	41,41414261	10,29553509	22,30953979
	44,94949341	10,29553509	22,30953979
	48,48484802	10,29553509	22,30953979
	52,02020264	10,29553509	22,30953979
	55,05050659	10,29553509	22,30953979
	58,58585739	10,29553509	22,30953979
	37,87878799	-14,05599594	35,1728363
	41,41414261	-14,05599594	35,1728363
	44,94949341	-14,05599594	35,1728363
	48,48484802	-14,05599594	35,1728363
	52,02020264	-14,05599594	35,1728363
	55,55555725	-14,05599594	35,1728363
	59,09090805	-14,05599594	35,1728363
	37,87878799	-37,6861763	32,83775711
	41,41414261	-37,6861763	32,83775711
	44,94949341	-37,6861763	32,83775711
	48,48484802	-37,6861763	32,83775711
	52,02020264	-37,6861763	32,83775711
	55,55555725	-37,6861763	32,83775711
	59,09090805	-37,6861763	32,83775711
	62,12121201	-37,6861763	32,83775711
	31,31313133	35,72780228	19,29811287
	31,81818199	32,14982605	18,56517601
	32,32323074	28,08279228	17,2622776
	32,8282814	24,50481796	16,52934074
	33,33333206	20,92684174	15,79640293
	33,33333206	16,83404732	15,03630352
	33,83838272	13,25607109	14,30336666
	34,34343338	9,703857422	13,02762985
	36,36363602	-5,61192131	9,498757362
	36,86868668	-9,189897537	8,76581955
	37,37373734	-12,76787281	8,03288269
	41,91919327	-29,47525406	44,69830322
	42,42424393	-27,77622032	41,522995
	42,42424393	-25,53660393	37,83205414
	42,42424393	-23,29698753	34,14110947
	42,42424393	-21,5979538	30,96580315
	42,92929459	-19,89892006	27,79049683
	43,43434525	-17,65930367	24,09955406
	43,43434525	-15,41968822	20,4086113
	44,44444275	-7,56815052	7,218910694

	44,44444275	-5,843354225	3,500804901
	44,94949341	-4,144320011	0,2660424411
	7,575757504	38,50885773	-7,310443878
	11,11111069	38,50885773	-7,310443878
	14,64646435	38,50885773	-7,310443878
	28,78787804	37,47921753	-7,369942665
	32,32323074	37,47921753	-7,369942665
	42,92929459	37,47921753	-7,369942665
	46,46464539	37,50498199	-7,964437008
	38,88888931	9,679813385	-32,81568146
	43,93939209	9,705575943	-33,41017914
	47,4747467	9,731338501	-34,004673
	51,01010132	9,731338501	-34,004673
	54,04040527	9,731338501	-34,004673
	37,87878799	8,752365112	-11,41389179
	41,41414261	8,752365112	-11,41389179
	44,94949341	8,752365112	-11,41389179
	48,48484802	8,752365112	-11,41389179
	52,02020264	8,752365112	-11,41389179
	55,55555725	8,752365112	-11,41389179
	40,40404129	-22,03462791	7,543955803
	43,93939209	-22,03462791	7,543955803
	47,4747467	-22,03462791	7,543955803
	51,01010132	-22,03462791	7,543955803
	54,54545593	-22,03462791	7,543955803
	58,08080673	-22,54944801	7,516793251
	61,61616135	-23,0642662	7,489630699
	65,15151215	-23,0642662	7,489630699
	24,74747467	-18,99637604	-14,20935345
	27,77777863	-18,99637604	-14,20935345
	31,31313133	-18,99637604	-14,20935345
	56,06060791	-18,99637604	-14,20935345
	59,59595871	-18,99637604	-14,20935345
	63,13131332	-18,99637604	-14,20935345
	26,76767731	-36,03738022	-2,08246541
	30,30303001	-36,03738022	-2,08246541
	33,83838272	-36,03738022	-2,08246541
	58,08080673	-36,03738022	-2,08246541
	61,61616135	-36,03738022	-2,08246541
	65,15151215	-36,03738022	-2,08246541
	25,25252533	-48,03237915	-11,11937237
	28,78787804	-48,0066185	-11,71386623

	32,32323074	-47,98085403	-12,30836105
	35,85858536	-47,98085403	-12,30836105
	39,39393997	-47,98085403	-12,30836105
	42,92929459	-47,98085403	-12,30836105
	46,46464539	-47,98085403	-12,30836105
	57,07070541	-47,98085403	-12,30836105
	60,10100937	-47,98085403	-12,30836105
	63,63636398	-47,98085403	-12,30836105
	31,31313133	37,06444168	67,25457001
	-43,43434525	5,145620346	65,57048798
	-50,50505066	5,145620346	65,57048798
	-32,32323074	-39,12887573	63,2345047
	10,10101032	44,27191925	67,63484192
**Subject 6**	-7,134299755	-42,50049591	57,78473282
	-19,20392418	-43,15734863	57,59541321
	-23,20805931	-43,37481308	57,53761673
	-27,19894028	-43,58733368	57,47647858
	-31,17069435	-43,79258728	57,40787888
	-35,1193428	-43,98826599	57,32848358
	-39,04243851	-44,1723938	57,23667145
	-42,93608475	-44,34420395	57,13200378
	-46,79809189	-44,50364685	57,01492691
	-50,62824249	-44,65150452	56,88721848
	-54,42936325	-44,78904724	56,75149536
	-58,20441818	-44,91782379	56,61045837
	-26,81278419	-10,77023602	41,67551041
	-30,84428787	-10,8328743	41,78424454
	-34,83999634	-10,89954185	41,88401794
	-38,79462051	-10,96922112	41,97245407
	-42,70418549	-11,04125977	42,04823303
	-46,56781387	-11,11513424	42,11129761
	-50,38840103	-11,18959141	42,16261292
	-54,16944885	-11,26371574	42,2037468
	-57,91590881	-11,33701229	42,23685455
	-61,63631058	-11,40723133	42,2649765
	-65,34082031	-11,47380733	42,29182816
	-7,578802586	-28,477705	60,2075119
	-11,613307	-28,69967079	60,18817902
	-15,63933849	-28,92657852	60,17541122
	-19,65329742	-29,15738487	60,16656876
	-23,65188599	-29,39138222	60,15845871
	-27,63339233	-29,62717628	60,14781952

	-31,59497643	-29,86272812	60,13152313
	-35,5328064	-30,09611893	60,10678101
	-39,44339752	-30,32525444	60,07173157
	-43,32550812	-30,54848289	60,02604675
	-47,17932892	-30,76473045	59,97048569
	-51,00416946	-30,97298813	59,90615845
	-54,80303955	-31,17240715	59,83502579
	-58,5802269	-31,36262894	59,75974655
	-30,35716248	-9,50676918	42,80586243
	-38,34791183	-10,01943684	42,7101059
	-42,26445007	-10,2820673	42,64037323
	-46,13801575	-10,5459547	42,55866241
	-49,96958542	-10,81012821	42,46559143
	-53,76077652	-11,07295418	42,36251831
	-57,51685715	-11,33403587	42,25198364
	-11,14368057	-22,13619614	42,13246918
	-15,25894356	-22,29953384	42,07577515
	-19,36707497	-22,46767616	42,02301407
	-23,4630146	-22,63978958	41,97179031
	-27,5405159	-22,8151226	41,91977692
	-31,59140587	-22,99283218	41,86399078
	-35,60720062	-23,17123985	41,80112457
	-39,58269119	-23,34862709	41,72914505
	-43,514431	-23,52413177	41,64693451
	-47,3993187	-23,69555092	41,55272293
	-51,23698425	-23,86157608	41,44604111
	-55,03230286	-24,02185822	41,32883453
	-58,79256439	-24,17590904	41,20333099
	-62,52521133	-24,32320404	41,07196426
	-13,13965416	-25,4019165	47,58850479
	-17,22200966	-25,69094849	47,43644714
	-21,29579926	-25,98491669	47,28803253
	-25,3567543	-26,28236389	47,14053345
	-29,39871025	-26,58171844	46,99089432
	-33,41412354	-26,88158798	46,83592606
	-37,39704132	-27,18009377	46,672966
	-41,34276962	-27,47532654	46,4993248
	-45,24663162	-27,76541138	46,31322861
	-49,10715485	-28,04877281	46,11507797
	-52,92658234	-28,32379532	45,90576553
	-56,70986557	-28,5899334	45,6866188
	-60,4618988	-28,84727859	45,45992661

	-64,1907959	-29,0955925	45,22875977
	-33,14418793	-29,38412285	37,76822281
	-37,15369797	-29,7822094	37,98339081
	-41,11790085	-30,17651558	38,18972778
	-45,03279495	-30,56444168	38,38517761
	-48,89862823	-30,94420242	38,56882858
	-52,71702194	-31,31431389	38,74057007
	-56,49193573	-31,67411804	38,90105057
	-60,23192596	-32,02336121	39,05229568
	-63,94731903	-32,36213684	39,19721985
	-34,02857971	-0,1057848334	25,69182014
	-37,9793663	-0,1753714979	25,42968559
	-41,87847137	-0,2520178258	25,16045761
	-45,72771835	-0,3351068795	24,88446808
	-49,52769852	-0,4231135845	24,60168076
	-53,28174973	-0,514338553	24,31262398
	-56,99721146	-0,6072152853	24,01922226
	-60,68424606	-0,6986292005	23,72379112
	-34,85242462	-14,86538601	20,38096428
	-38,84609985	-15,188941	20,33243561
	-42,78647995	-15,51310158	20,27897644
	-46,674263	-15,83699989	20,22086334
	-50,51096725	-16,15894699	20,15842056
	-54,30023193	-16,47781563	20,09226418
	-58,04978943	-16,79334831	20,02366066
	-61,77041245	-17,10412598	19,95406914
	-33,99305725	-25,66715813	28,29884148
	-38,0105896	-25,95822716	28,14204025
	-41,98023605	-26,24667168	27,97886848
	-45,89843369	-26,53122139	27,80869293
	-49,76190186	-26,81019783	27,63118172
	-53,57518387	-27,08239555	27,44641685
	-57,34774399	-27,34790421	27,25569153
	-61,08638	-27,60642624	27,06111717
	-64,80134583	-27,85836029	26,86504173
	-68,50604248	-28,1043644	26,67007256
	0	67	-4,212999821
	0	-22,62000084	75

Supplementary Table 1: MNI coordinates of bipolar recordings for all subjects
